# On the Intestinal Mucous Membrane

**Published:** 1849-02

**Authors:** Handfield Jones


### RECORD OF MEDICAL SCIENCE.

### ANATOMY AND PHYSIOLOGY.

*On the Intestinal Mucous Membrane.* By Dr. HANDFIELD JONES.—The author directs attention to a layer of granular matter, enveloping nuclei, which lies immediately beneath the mucuous membrane of the intestines, and in contact with the basement membrane. This granular layer or "*substratum*" of the mucous tissue, which is well seen in the colon, forms, together with the nuclei above mentioned, a great part of each villus, filling up the whole space between the limitary membrane, and the lacteal and capillary vessels. This substratum is the seat of the black discoloration, which is not uncommon in the intestinal mucuos membrane; and is also the part principally affected in dysentery, in which it is often left bare by the disappearance of the mucous membrane, and is infiltrated by a plasma passing into imperfect cellular forms.

In the period of inactivity, the villi are semi-transparent; when absorption is going on, however, they are opaque from the presence of oily matter in the form of globules and granules of different sizes. The larger of these Dr. Jones believes to correspond with the absorbing cells described by Professor Goodsir, as formed during the process of absorption, and which are very inconstant in their number and size, being sometimes entirely absent in villi which are manifestly in a state of activity. The opacity caused by the absorption of the chyle may be also observed frequently to pervade the whole length of the villus, thus indicating that this function is carried on in every part, although probably more active, as described by Mr. Goodsir, at the summit. The active agents of absorption are believed by the author to be the nuclei distributed among the granular matter of the villus, and which, though obscured during the process by the presence of milky chyle are constantly visible in the inactive and semi-transparent villus. "It is well known now, that the formation of perfect cells is by no means to be regarded as essential to the exercise of the energy of nuclei, those fundamental and efficient parts of almost all cell formations. It is also known that the formation of perfect cells indicates a certain degree of permanence in the structure so formed, and that their contents are destined to be retained for a period, to undergo some elaborating change, not to be immediately yielded up; while, on the other hand, the non-completion of cells indicates that the process is of a rapid character, and not intended to produce any considerable change in the material acted on. Remembering these facts (of the general truth of which there cannot be much doubt,) it will be admitted, perhaps, as highly probable, that the nuclear corpuscles of the grauular basis of the villi exert an attraction on the chyme by which they are surrounded, and draw it continually into the substance of the villus, from whence it is rapidly conveyed away by the efferent lacteal."

The author agrees with Professor Weber, that the shedding of their epithelium is not *necessary* to enable the villi to perform their functions. He has seen the villi clad with their epithelium when the lacteals were filled with chyle; nevertheless it is certainly common to find them divested of this covering when absorption is most actively present. Dr. Jones corroborates the observation of M. Lacauchie, that the villi are subject to retraction and thickening, under the influence of circumstances which are not well understood, as they do not appear to be possessed of any contractile tissue as an element of their structure, and "the distension of their capillary plexus with blood would rather have a contrary effect." [We think, on the contrary, that this shortening and thickening of the villi is a very probable effect of vascular or any other distension.]

Dr. Jones has examined with great care the solitary and aggregated glands of the intestine in the human subject, as well as in the rabbit and dog. The patches, he considers, like most other anatomists, to be merely aggregations of solitary glands. The depressions in the aggregated glands do not correspond to any distinct open mouths of follicles, but seem to be produced by the absence of villi from those parts of the mucous surface. The structure of the solitary and aggregated glands is very similar; they are situated in the substratum, below the basement membrane, and covered by a plexus of capillary vessels. On making a vertical section of one of the solitary glands of the human intestine, it is seen by the microscope to consist of masses of nuclear granules, "which, for the most part, are solid, not including a distinct cavity, and not contained in any definite follicular envelope; they lie at various depths; the larger are in contact with the surface, the mucous membrane, with its rows of vertical follicles, having disappeared above them; the smaller lie unquestionably beneath the mucous surface, and I feel quite assured, have no orifice of communication by which their contents might escape into the intestinal cavity; even pretty strong pressure does not evacuate the contents of the smaller masses, while it sometimes produces this effect on the larger, which more closely adjoin the surface. The form of these masses varies a good deal; often they are considerably flattened, usually, however, more or less globular—their upper portion being always convex, and tending to approach the surface; when it reaches this, the mass appears to become more or less completely evacuated, and a shallow depression may then result; this, however, is but rarely seen. In the cæcum of the dog, the solitary glands are more or less prominent on the surface, and exhibit a very distinct appearance of a central orifice. When macerated in acetic acid, they appear as circular spots about the size of a large pin's head, rather flattened, and with perfectly defined margins. In vertical sections through the central orifice the mucous membrane is seen to dip down, and become gradually thinner; sometimes it appears to be perforated at the bottom of the depression; at others is continued plainly across.

The gland itself consists of a solid mass of nuclear corpuscles, with a little granular matter. It is contained in a kind of capsule, which seems to belong to the submucous tissue; at the bottom of the depression, the mass comes in contact with the thin mucous membrane, if it exists, or with the orifice, if it be absent; but can rarely be made to escape even by strong pressure. It does not appear that these glands can be regarded as true follicles, their capsule is not continuous with the basement membrane; their contents are not epithelial particles lining the wall, but a solid mass of nuclei; and, lastly, the existence of an orifice in them does not seem constant, whether evidence of it be sought for by minute examination, or by observing the effect of pressure upon their mass. In the rabbit the long and wide appendix cæci has its mucous lining greatly thickened by a layer of masses consisting of nuclear granules; these are of elongated conical form; their apices reach to the surface, and lie in fossulæ formed by septal folds of mucous membrane; over their surfnce a capillary plexus is spread supplied by the long vessels which run up from below; they appear to be quite solid, and their apex is certainly not perforated, but in some instances appears to be invested by a distinct homogeneous membrane. In all these cases, it is worthy of remark that the masses of nuclear granules are affected in a peculiar manner by acetic acid; instead of rendering them transparent, it makes them much more opaque, so that their outlines become extremely distinct even to the naked eye. This circumstance, as well as the marked difference between their contents, and the epithelium of any glands or follicles, is very characteristic of them, and tends to prove that they are not mere follicular involutions of the mucous surface, but superadded structures designed for some special but unknown function."

The aggregated and solitary glands would thus appear to be, according to Dr. Jones' observations, an increased development in particular localities, of the nuclei and granular matter which he described as forming the general substratum of the mucous membrane. In the small intestine, where, he says, there are in health few or no *solitary* glands, such as exist in the colon, the bodies which in the diseased state are described as such are apparently adventitious structures, and are composed of nuclei and granules similar to those of the gland, but not inclosed in any capsule. The author believes these to be analogous to pimples of the skin, and, like them, capable of disappearing by absorption.—*Medical Gazette*, November 17, 1848.

